# In Situ Fluorine Doping of FZO Films by Atomic Layer Deposition

**DOI:** 10.1021/acsomega.5c10192

**Published:** 2026-07-19

**Authors:** Meryem Tunckanat, Bilge Imer

**Affiliations:** † Micro and Nanotechnology Department, 52984 Middle East Technical University, Ankara 06800, Türkiye; ‡ Department of Metallurgical and Materials Engineering, 52984 Middle East Technical University, Ankara 06800, Türkiye

## Abstract

In optoelectronic devices, fluorine-doped semiconductors have been used widely. Atomic layer deposition (ALD) is one of the main processes integrated into semiconductor manufacturing and a perfect candidate to obtain conformally covered films for complex geometries at low temperatures on a budget. Due to the lack of a proper fluorine source, fluorine doping with ALD has been a challenge. In this study, fluorine-doped ZnO (FZO) thin films by ALD were successfully achieved, demonstrating the efficiency and practicality of a new homemade precursor mixture: ammonium fluoride/water (NH_4_F/DI) solution. The fluorine incorporation mechanism was investigated and discussed in detail with respect to growth conditions. The effects of ALD growth temperature (160–200 °C), canister temperature (RT, 40 °C, and 50 °C), and concentration of NH_4_F/DI water mixture (20–40%) on fluorine doping, crystallinity, atomic-scale interactions, and electrical and optical properties were investigated. For the fluorine doping conditions identified as 180 °C growth temperature with a 30% NH_4_F/DI water mixture and a 40 °C canister temperature, a fluorine incorporation of 2.62 at. % F was achieved.

## Graphical abstract

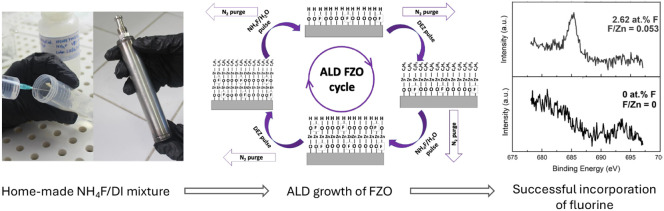

## 1 Introduction

Metal oxide semiconductors exhibit a wide range of optical and electrical properties, governed by their intrinsic point defect concentrations and dynamics.^1–3^ These materials are commonly employed as transparent conductive oxides (TCOs) in optoelectronic applications. Due to their versatility, TCOs play a critical role in technologies such as sensors, solar cells, LEDs, and flat-panel displays.^4–9^ Although indium tin oxide (ITO) remains the dominant commercial TCO owing to its superior optoelectronic performance, the development of indium-free alternatives has become imperative due to indium’s toxicity, scarcity, and high cost.^10,11^ To meet the growing demand in optoelectronics, researchers must explore new materials and enhance existing TCOs using low-cost, chip-manufacturing-compatible deposition techniques like atomic layer deposition (ALD).

Zinc oxide (ZnO) is an intrinsically n-type semiconductor that has gained prominence in electronics, optoelectronics, biomedical applications, and sensing technologies due to its exceptional optoelectronic and thermal properties. These include a direct wide bandgap (3.37 eV), high electron mobility (1–200 cm^2^/V·s), large exciton binding energy (60 meV), robust radiation resistance, and thermal stability.^12–15^ Additionally, ZnO is abundant, low-cost, and environmentally benign.^10,16^ Recent research has focused on doping strategies to further improve ZnO’s conductivity, optical transparency, and overall device performance.

Dopants in ZnO can be categorized into two groups: trivalent metal cations (Al, Ga, B) and halogen anions (F, Cl).^17–20^ Among these, fluorine has emerged as one of the most effective dopants for oxide semiconductors, significantly enhancing ZnO’s properties. Fluorine-doped ZnO (FZO) exhibits such improved characteristics that it has become a promising material not only for transparent conductive oxide (TCO) applications but also for gas sensors, UV photodetectors, and photocatalytic devices.^21^ Nevertheless, the underlying mechanism of fluorine doping in metal oxides remains a subject of ongoing debate within the scientific community.

The ionic radii of fluorine (0.136 nm) and oxygen (0.140 nm) ions are nearly identical, while the electronegativity of fluorine (3.98) is higher than that of oxygen (3.44). Therefore, some researchers suggest that fluorine easily substitutes for oxygen sites.^22^ The substitution of fluorine ions for oxygen increases the free electrons in the system and reduces the electrical resistance of the oxide-based films. Furthermore, fluorine doping can passivate intrinsic oxygen-related defects.^23^ However, density functional theory (DFT) calculations show that fluorine antisites (F_O_) act as deep donors and cannot provide free electrons to the system. Fluorine primarily passivates dangling bonds on surfaces and eliminates oxygen-related defects. Also, at high fluorine concentrations, interstitial fluorine (F_i_) forms a deep acceptor, compensating for n-type doping.^24^

FZO thin films have been grown using chemical methods, such as solution-based spray pyrolysis and chemical vapor deposition (CVD), and various physical vapor deposition (PVD) techniques.^23,25–28^ The resistivity values of the films deposited by spray pyrolysis have high electrical resistivities.^26,29^ Thus, the films produced by the spray pyrolysis method cannot provide the required electrical properties for TCO applications. There have been remarkable reports on PVD-grown FZO films with resistivities lower than 8 × 10^–4^ Ω·cm and transmittance in visible light higher than 85% ^23,27^ and on CVD-grown FZO films with a resistivity of 4 × 10^–4^ Ω.cm and mobility of 40 cm^2^ V^–1^ s^–1^.^26^ However, these methods present limitations: PVD requires postdeposition treatments (e.g., oxidation, annealing) and lacks conformal coverage, and CVD typically demands high deposition temperatures (>450 °C), restricting substrate compatibility. In addition, the investment and operational costs of PVD and CVD are higher than those of ALD.

ALD is a vapor-phase thin-film deposition technique based on self-limiting surface reactions. Unlike conventional CVD, ALD offers exceptional capabilities, including conformal coverage on high-aspect-ratio and complex 3D structures, atomic-level thickness control (subnanometer precision), excellent uniformity across large-area substrates, and low-temperature processing compatibility. These unique advantages make ALD particularly suitable for micro- and nanoelectronic fabrication, where precise, conformal coatings are essential, with a wide range of substrate choices.^30^

Despite the advantages of ALD, *in situ* fluorination remains challenging due to the lack of suitable fluorine precursors. While TaF_5_ and TiF_4_ have been explored as fluorine sources,^31,32^ their high metal impurity content (Ta or Ti) makes them unsuitable for depositing high-purity fluorine-doped metal oxides. To address this, Park et al. proposed using a hydrofluoric acid (HF)/deionized (DI) water mixture as an alternative fluorine source for ALD-grown FZO films.^33–35^ However, HF’s extreme corrosiveness—which may damage the ALD system, especially from precursors to chamber components—necessitates extensive modifications, such as Teflon-coated piping, as also mentioned in these studies. Furthermore, precise doping control is difficult, as HF concentrations must be kept very low (0–1%) to mitigate its aggressive etching behavior. A promising alternative is ammonium fluoride (NH_4_F), which decomposes into HF *in situ*, so it requires no system modifications, even at high concentrations. As detailed later, a 0.125% HF/DI solution exhibits similar etching strength to a 10% NH_4_F/DI solution, and NH_4_F/DI enables finer doping control across a wider concentration range. Once this cheap, safe, and easy-to-handle homemade precursor, NH_4_F/DI mixture, is demonstrated as a potential candidate, FZO production will also be economically much more feasible to produce by ALD compared with other counterparts like PVD and/or CVD.

In this study, the deposition of ALD-grown FZO films was achieved, demonstrating the practicality and efficiency of a new homemade precursor—a mixture of NH_4_F/DI water solution. The fluorine incorporation mechanisms, along with the compositional and structural properties of FZO films, were investigated for different growth conditions. Also, the effect of fluorine doping concentration on the optical and electrical properties of FZO films was investigated. Films were produced at various deposition temperatures between 160 °C and 200 °C, with 20–40% NH_4_F/DI mixture concentration, and canister temperatures set to room temperature, 40 and 50 °C.

## 2 Materials and Methods

In this study, FZO films were grown over silicon (p-type SSP (100)) and quartz wafers using the OkyayTech ALD reactor. The wafers were ultrasonically (US) cleaned in acetone (≥99.9%), ethanol (≥99.9%), and deionized (DI) water for 2 min each. To remove the native oxide layer, silicon wafers were soaked in a 4% hydrofluoric acid (HF)/DI water solution for 2 min prior to growth. Diethylzinc (DEZ, Sigma-Aldrich, Inc.) as a zinc source and NH_4_F/DI water solutions with varying concentrations of NH_4_F (20–40%) in DI water were utilized as fluorine and oxygen sources. As a carrier gas, N_2_ (≥99.99%) was used with a 20 sccm flow rate. The pulse times for DEZ and NH_4_F/DI water solution were selected as 100 ms to achieve surface saturation. The nitrogen purge time was chosen as 10 s. These pulse and purge times were determined based on well-established studies on the effect of pulse/purge times on surface saturation and growth rates.^36,37^ The ALD cycle for film growth was established as “Pump NH_4_F/DI water solution 100 ms/Purge for 10 s/Pump DEZ 100 ms/Purge 10 s.” This growth cycle was looped for 300 counts. The temperature of the canister containing the NH_4_F/DI water solution was maintained at three different temperatures during the growth: room temperature, 40 °C, and 50 °C. Films were grown at various temperatures between 160 °C and 200 °C. A summary of all experiments is shown in Table 1. Samples are named based on the deposition temperature, NH_4_F concentration, and precursor temperature, in that order, and separated by slashes (e.g., FZO (deposition temperature/%NH_4_F concentration/precursor temperature)).

**Table 1:** Sample IDs and their Deposition Conditions

Sample I.D.	Temperature (°C)	NH_4_F concentration (%)	Precursor Temperature (°C)	Cycle
FZO(160/20/50)	160	20	50	300
FZO(160/30/50)		30		
FZO(160/40/50)		40		
FZO(180/20/50)	180	20	50	
FZO(180/30/*RT*)		30	RT	
FZO(180/30/40)		30	40	
FZO(180/30/50)		30	50	
FZO(180/40/50)		40	50	
FZO(200/20/50)	200	20	50	
FZO(200/30/50)		30		
FZO(200/40/50)		40		

## 3 Characterization

The crystallinity and the presence of phases were determined by Grazing Incidence X-ray Diffraction (GIXRD, Rigaku Ultima-IV) using Cu Kα (α = 1.5418 Å) radiation at 40 kV and 30 mA. Film thicknesses were determined by spectroscopic ellipsometry using a Semilab Spectroscopic Ellipsometry Analyzer (SEA). The optical model was fitted using a Cauchy dispersion model at an incidence angle of 70°. The uncertainty in thickness values corresponds to the fitting error obtained from the ellipsometric regression as ±1 nm.

Time-of-flight secondary ion mass spectroscopy (TOF-SIMS) was used to analyze the distribution of fluorine atoms in the films. Mass spectra were carried out by a 4000 V pulsed O_2_ gun with 100 nA/V detector sensitivity for zinc (^64^Zn) and silicon (^28^Si), operating at a chamber pressure of 1.3 × 10^–7^ Torr. Mass spectra of silicon (^28^Si), carbon (^12^C), oxygen (^16^O), and fluorine (^19^F) were obtained by a 5000 V pulsed Cs gun with 10 nA/V detector sensitivity, operating at a chamber pressure of 2.7 × 10^–8^ Torr. Fluorine doping concentration was detected by X-ray Photoelectron Spectroscopy (XPS) using a monochromatic Al Kα (1486.6 eV) source with a 400 μm spot size. XPS measurements were taken from the surface of the films in high-resolution (HR) mode with a 50 ms dwell time. The pass energy for the survey scan was 200 eV with a 1 eV step size, and for the partial scan, it was 50 eV with a 0.1 eV step size. Partial scans were done for F 1s, Si 2p, C 1s, Zn 2p, and O 1s spectra. Prior to measurement, etching was applied to the surface with 1000 eV etch energy for 120 s. Quantitative analysis was conducted using CasaXPS software, applying deconvolution and integration of core-level peaks after Shirley background subtraction. Elemental atomic percentages were determined using Relative Sensitivity Factors (RSFs) provided within the software. No explicit correction was applied for the angular distribution or the inelastic mean free path, as the measurements were conducted at normal emission geometry (90° takeoff angle).

Resistivity measurements were performed on all samples using a Signatone Pro-4 four-point probe system coupled with a Keithley 2400 source meter. The measurements were carried out using a standard collinear four-probe geometry with a probe spacing of 1 mm, where current was applied through the outer probes and voltage was measured across the inner probes. The Hall mobility, carrier concentration, and resistivity of the films were measured only for the lowest resistivity sample by a Hall measurement system using the van der Pauw method at room temperature under a 2000 G magnetic field. Aluminum was coated by an evaporation technique (around 35 nm) onto the edges of films grown on a quartz substrate.

The transmittance of the films was measured in the 190 and 1000 nm region by UV–vis measurement. The Haacke figure of merit (Φ_TC) was calculated using Φ_TC = *T* ^10^/R_s, where *T* is the average transmittance in the 400–700 nm range.

## 4 Results and Discussion

### 4.1 NH_4_F/DI Water Mixture as Fluorine and Oxygen Precursors

The precursors used in ALD should satisfy certain key criteria, including volatility, reactivity, thermal stability, purity, affordability, etc. In this work, NH_4_F/DI water solution was employed as a combined fluorine and oxygen source. This solution has several advantages over HF, such as being a weaker acid, practical to handle, cost-effective, and having optimal volatility with a vapor pressure close to water.

To find the relative damage and etch rate of NH_4_F in comparison with HF, ALD-grown ZnO films on Si substrates with 100 nm thickness were etched by different NH_4_F/DI water concentration solutions, varying as 1%, 5%, and 10%, for one minute. After that, the etch depth was measured by a DEKTAK depth profiler to calculate the etch rate, as shown in Table 2. According to a study in the literature,^38^ the etch rate of HF for aluminum zinc oxide (AZO) films (with about 3% Al content) on Si substrates was investigated using HF/DI water solutionsof varying concentrations (0.125%, 0.25%, 0.5%, and 1%), as also shown in Table 2. The etch rates of various HF solutions for AZO films, compared with the etch rates of various NH_4_F solutions for ZnO films, are given in Table 2. Accordingly, the concentration of a 0.125% HF/DI water solution has the same etch rate as a 10% NH_4_F/DI water solution on ZnO films.^38^ Thus, as a new fluorine-based precursor, NH_4_F is much less damaging to handle for users and the ALD system, especially prior to arriving at the chamber for in situ fluorine doping, and it has a wider range of tunability.

**Table 2:** Etch Rates of NH_4_F on ZnO Films (This Study), and the Study for Etch Rate of HF on AZO Films^38^

Film	NH_4_F concentration (%)	Etch rate (nm/s)	Film^38^	HF concentration (%)^36^	Etch rate (nm/s)^36^
ZnO	1	0.5	AZO	0.125	1.1
	5	0.8		0.25	1.1
	10	1.1		0.5	1.2
				1	1.4

Utilizing this advantageous NH_4_F/DI mixture precursor, the growth mechanism of FZO via ALD can be understood by identifying possible half-reactions.

The NH_4_F dissolves in water as^39^

1 $$NH_{4}F+H_{2}O↔N{H_{4}}^{+}+F^{-}$$

2 $$N{H_{4}}^{+}+H_{2}O↔NH_{3}+H_{3}O^{+}$$

The possible half-reactions after exposure to DEZ and NH_4_F/DI water mixture in ALD are as follows:

-For DEZ Pulse

3 $$Si‐OH^{*}+Zn{\left(CH_{2}CH_{3}\right)}_{2}\to Si‐O‐ZnCH_{2}C{H_{3}}^{*}+C_{2}H_{6}$$

-For NH_4_F/DI mixture Pulse

4 $$\mathrm{Si}‐O‐\mathrm{ZnC}H_{2}C{H_{3}}^{*}+NH_{3}\to \mathrm{Si}‐O‐Zn‐N{H_{2}}^{*}+C_{2}H_{6}$$

5 $$\mathrm{Si}‐O‐ZnCH_{2}C{H_{3}}^{*}+H_{2}O\to \mathrm{Si}‐O‐ZnOH^{*}+C_{2}{H_{6}}^{-}$$

6 $$\mathrm{Si}‐ZnCH_{2}C{H_{3}}^{*}+F^{‐}\to \mathrm{Si}‐O‐ZnF^{*}+C_{2}H_{6}$$

Among these, eqs 4 and 5 are thermodynamically more favorable than [Disp-formula eq6], as supported by both experimental studies and first-principles calculations.^6,7,33,40^ This preference leads to the dominant formation of Zn–OH and Zn–F surface species.

Fluorine incorporation via F^–^ is particularly favored due to the strong Zn–F bond formation energy, which lowers the Gibbs free energy of the reaction path.^24,33^ On the other hand, Zn–NH_2_ formation is less likely due to higher formation energy and possible detrimental effects on n-type conductivity, as reported in previous studies.^40^

### 4.2 The Effect of Canister Temperature

The effect of canister temperature on fluorine doping concentration was experimented for a growth temperature of 180 °C and a 30% NH_4_F/DI concentration. The precursor canister temperature was systematically varied across three set points: room temperature (RT, ∼25 °C), 40 °C, and 50 °C (see Table 1). The effect of changes in canister temperature on crystallinity, fluorine concentration, incorporation depth, and stoichiometry-defect dynamics was investigated. All XRD data were identified based on the reference peaks given in Figure 1.

**Figure 1:**
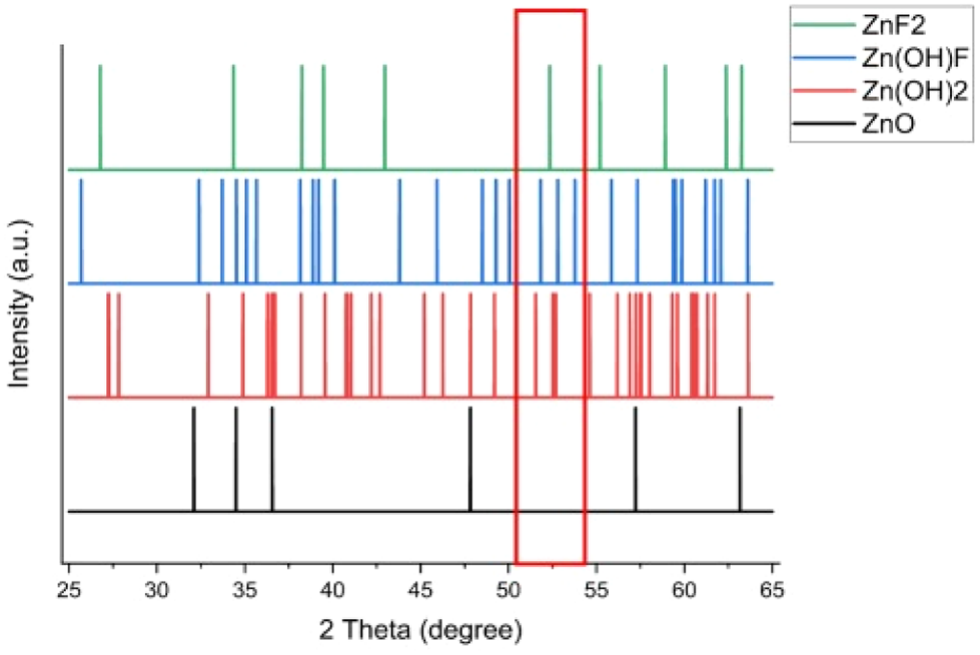
XRD patterns for wurtzite ZnO are based on JCPDS card no. 36–1451, and ZnF_2_, Zn­(OH)­F, Zn­(OH)_2_ peaks are identified based on peak matching using the QualX software and the Crystallography Open Database (COD), specifically highlighted for the 50°–55° range.

The XRD pattern of these samples is shown in Figure 2. In the same figure, the crystalline sizes for (100), (002), (411), (123), and (212) peaks are indicated along with the peak positions. The crystalline size (*D*) was calculated by using Debye–Scherrer equation (eq 7). *K* is the Scherrer constant (taken as 0.9), λ is the X-ray beam wavelength (0.15406 nm), β is the full width at half-maximum (fwhm) of the corresponding peak (in radians), and θ is the Bragg angle (degree).^41,42^

7 $$D=\frac{K\lambda }{\beta \;\cos\;\theta }$$

**Figure 2:**
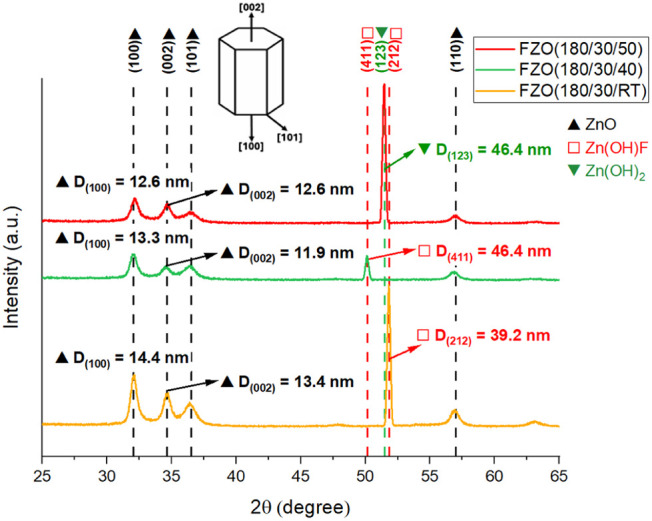
XRD patterns for samples with increasing canister temperatures from bottom to top (RT, 40 °C), and 50 °C) at a growth temperature of 180 °C and 30% NH_4_F/H_2_O concentration.

Figure 2 shows that all samples exhibit a dominant diffraction peak at approximately 51°. This observation can be attributed to the formation of ZnOHF compounds at high NH_4_F/DI water concentrations, facilitated by fluorine’s high electronegativity. According to ref 43, ZnOHF demonstrates remarkable thermal stability up to ∼390 °C, which stems from its hydrogen-bonding network.

The XRD analysis revealed a temperature-dependent crystallographic evolution. For the canister held at room temperature, the (212) plane of ZnOHF was dominant, with a calculated grain size of 39.2 nm. For a canister temperature of 40 °C, the preferred orientation shifted to the (411) plane of ZnOHF, with a reduced grain size of 36.4 nm (vs 39.2 nm for the (212)). For 50 °C canister temperature, the dominant peak appeared at 51.4°, corresponding to the (123) plane of Zn­(OH)_2_ with a grain size of 46.4 nm. The grain size for the (002) orientation decreased from 13.4 to 11.9 nm as the canister temperature increased from RT to 40 °C. This shift suggests a change in preferred crystallographic orientation due to altered fluorine incorporation or bonding dynamics at this temperature. Fluorine atoms could have preferred to form ZnOHF at a canister temperature of 40 °C instead of passivating oxygen defects, resulting in decreased grain size. The absence of a peak around 52° in the 40 °C sample indicates the suppression of both Zn­(OH)_2_ and the (212) ZnOHF orientation. This could be due to fluorine atoms favoring the formation of the (411) ZnOHF under this condition, preventing the crystallization of phases that normally contribute to peaks near 52°. Then, the (002) grain size increased from 11.9 to 12.6 nm with increasing canister temperature from 40 to 50 °C. In the 50 °C sample, fluorine atoms passivated oxygen defects, resulting in increased grain size and crystallinity.

Figure 3 shows the compositional analysis carried out by XPS and the CASA XPS analysis of the O 1s and Zn 2p_3/2_ regions.

**Figure 3:**
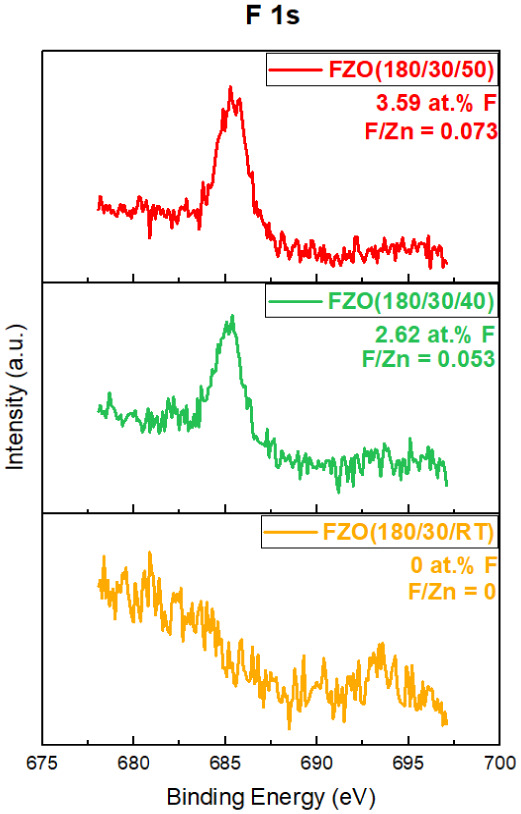
HR-XPS F 1s surface measurement peaks with increasing canister temperatures from bottom to top (RT, 40 °C, and 50 °C) at a growth temperature of 180 °C and 30% NH_4_F/H_2_O concentration.

In Figure 3a, the F/Zn ratio increased with increasing canister temperature, as expected. The vapor pressure of species in the precursor bottle increases with increasing temperature. Lighter and smaller atoms and molecules gain more momentum with increased thermal energy. As shown in eqs 1 and 2, the relative presence of fluorine atoms was expected to increase with an increase in temperature. In HR-XPS measurements, the O 1s peak consists of three peaks: OI, OII, and OIII. The percentage of the OI area indicates metal oxide (ZnO) bonding, the OII area indicates oxygen vacancies (Vo), and the OIII area indicates hydroxide (−OH). The Zn 2p_3/2_ peak is composed of Zn^2+^, ZnOH, and Zn^0^ (zinc interstitial) areas. The ratios of M-O/O_total_ (M-O%), V_oc_/O_total_ (V_oc_%), −OH/O_total_ (−OH%), Zn^0^/Zn_total_ (Zn%), Zn^2+^/Zn_total_ (Zn^2+^%), and ZnOH/Zn_total_ (ZnOH%) are calculated with the CASA XPS program and shown in Figure 3b.

Accordingly, V_oc_% did not change much from room temperature (0 at. % F) to 40 °C (2.62 at. % F). M-O%, Zn%, and Zn^2+^% decreased, while OH% and ZnOH% increased for 0 and 2.62 at. % F. This increase in OH% can be related to zinc atoms bonding to OH instead of only O, and fluorine ions being attached to Zn­(OH)_2_ to form ZnOHF instead of filling oxygen vacancies. This can be supported by the change in the (212) plane to the (411) plane orientation of ZnOHF for 40 °C in Figure 2.

For the change in canister temperature from 40 °C (2.62 at. % F) to 50 °C (3.59 at. % F), M-O%, OH%, and Zn­(OH)_2_ increased, while V_oc_%, Zn%, and Zn^2+^% decreased. For the 50 °C canister temperature, zinc atoms preferred to bond with both oxygen and OH groups, but fluorine ions preferred to fill oxygen vacancies and bond with zinc interstitials. The XRD pattern of the 50 °C canister temperature supports this argument; the Zn­(OH)_2_ peak ((123)) was observed instead of ZnOHF ((212) or (411)), and the reason behind that was fluorine occupying oxygen vacancies and bonding with zinc interstitials.

The fluorine distribution in films was measured by SIMS depth analysis. In addition to fluorine, carbon, oxygen, zinc, and silicon elements were also measured. The film thicknesses were determined by marking the position where the silicon signal picked up. The marker indicating film thickness was placed where the silicon signal picked up from the substrate. There was fluorine diffusion into the silicon substrate. Fluorine atom counts were normalized with respect to the film surface and compared for three different canister temperatures (RT, 40 °C, and 50 °C). The fluorine count values are arbitrary and comparable among samples rather than being absolute at.%. Therefore, relative trends indicate that the fluorine distribution depth and the fluorine concentration increased with an increase in canister temperature, as shown in Figure 4.

**Figure 4:**
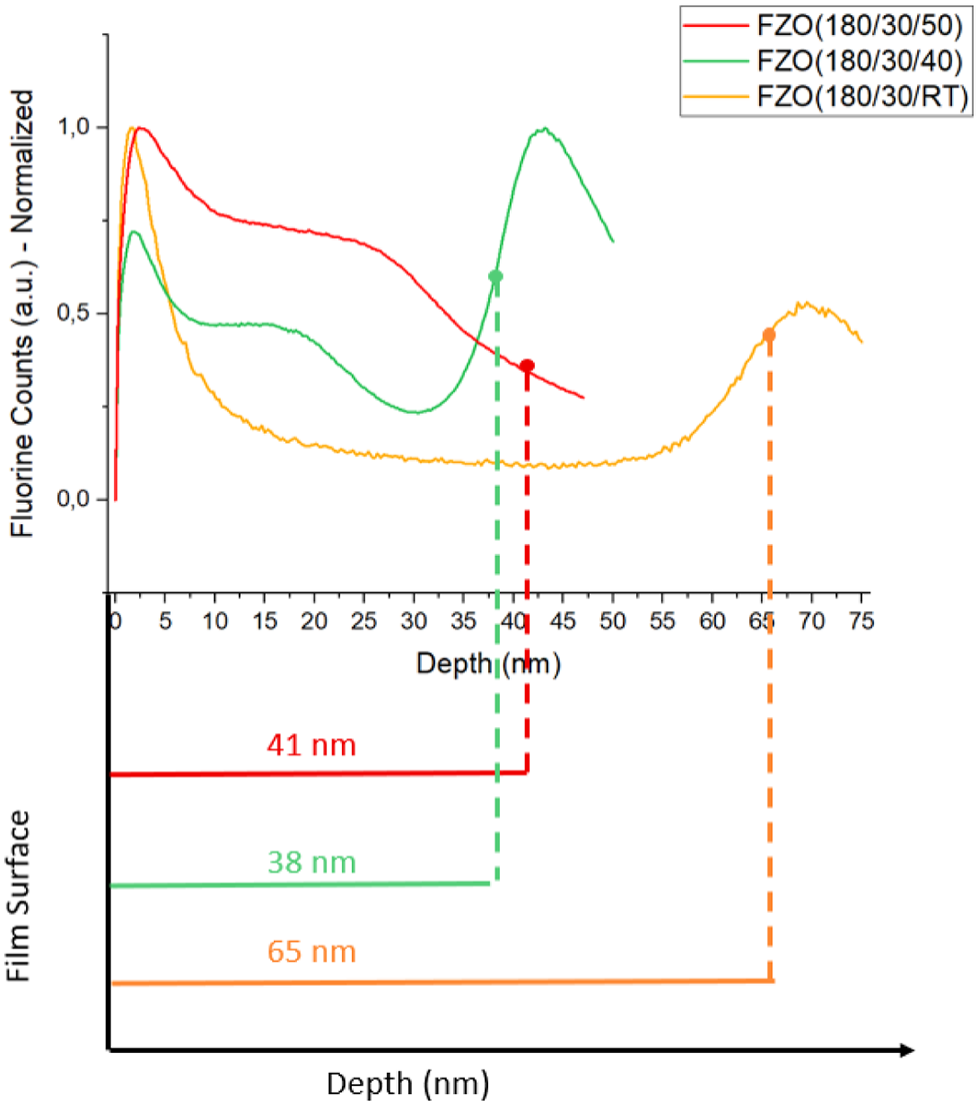
SIMS depth analysis for samples with increasing canister temperatures of RT, 40 °C, and 50 °C at a growth temperature of 180 °C and 30% NH_4_F/H_2_O concentration. The fluorine counts along the film depth are normalized with respect to the film surface.

The results in Figure 4 demonstrate a clear correlation between precursor canister temperature and fluorine incorporation efficiency. Increasing the canister temperature from RT to 50 °C enhanced fluorine doping in two key ways: enhanced precursor delivery and modified growth kinetics. Elevated temperatures increase NH_4_F/DI solution vapor pressure,^44^ improve precursor transport to the surface, and enable deeper fluorine penetration into the ZnO matrix. Also, the reactivity of the gas increased with canister temperature, and more gas species could interact with the surface, resulting in a reduced growth rate. The growth per cycle (GPC) was 1.3 Å per cycle on average. This value is slightly lower than the ranges given in the literature for ZnO films. The growth rates of ZnO in the literature are in the range of 1.5–2 Å per cycle, depending on growth orientation, (100) or (002),^45^ and growth temperature.^46^ The reduced growth rate and increased reactivity together led to obtaining more fluorine doping at a greater depth.

The optical transmittance of FZO films exhibits a strong dependence on the Zn/[O + F] ratio. Increased zinc content promotes light scattering through the formation of zinc-related defect centers and the development of substoichiometric phases. This results in significant transmittance reduction, as reported.^47–49^ Figure 5 presents the optical transmittance spectra of FZO films grown at different precursor canister temperatures. The average visible-range transmittance (400–700 nm) shows a clear temperature dependence. At room temperature, the average transmittance was around 81%. At 40 °C, 80% visible-range average transmittance (attributed to the increased Zn/(O + F) ratio) was observed, and at 50 °C, 89.4% visible-range average transmittance (resulting from the reduced Zn/(O + F) ratio) was obtained. The observed 11% visible-range average transmittance enhancement at 50 °C compared with the 40 °C samples demonstrates the significant impact of stoichiometry control through temperature-dependent fluorine incorporation.

**Figure 5:**
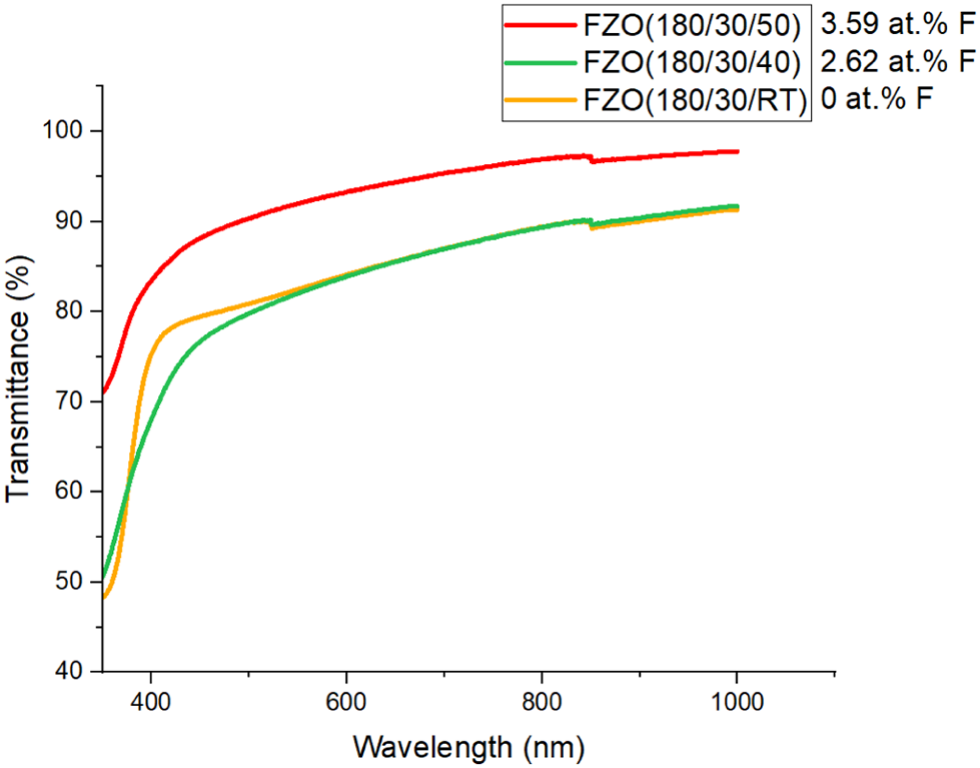
Transmittance percentages for samples with increasing canister temperatures of RT, 40 °C, and 50 °C at a growth temperature of 180 °C and 30% NH_4_F/H_2_O concentration.

#### 4.2.1 Mechanism Interpretation

An increase in canister temperature promoted fluorine incorporation, resulting in a direct correlation with the overall doping concentration due to increased fluorine flux with respect to the rest of the species in the NH_4_F/DI mixture. The fine-tuning of precursor temperature would significantly affect the final fluorine incorporation in ZnO films, keeping all other growth variables constant. The precursor temperature can also modulate the competing rates of film growth and fluorine diffusion to enhance the concentration homogeneity.

### 4.3 Effect of Growth Temperature and NH_4_F/DI Concentrations

This study examines FZO film growth via ALD across three substrate temperatures (160 °C, 180 °C, and 200 °C) using 300 ALD cycles for all depositions. The NH_4_F/DI water solution concentration was varied from 20% to 40%, as detailed in Table 1, while maintaining a constant precursor canister temperature of 50 °C throughout all experiments.

In literature, ALD growth temperatures for ZnO were studied between 100 and 200 °C in several studies.^50–52^ At a growth temperature of 200 °C, the ZnO films are produced as zinc-rich and have a (002) preferential growth orientation. As fluorine doping concentration increases, the preferential growth orientation changes from (002) to (100), as shown by Kang et al.^53^ In another study, at 140 °C, as the number of growth cycles increased from 300 to 1200, ZnO films exhibited a (002) peak-dominated growth orientation, while fluorine-doped ZnO films had a dominant (100) growth orientation.

The XRD patterns for varying NH_4_F/DI concentrations (20%, 30%, and 40%) for each growth temperature (160 °C, 180 °C, and 200 °C) are shown in Figure 6. For all NH_4_F/DI concentrations at all growth temperatures, as fluorine concentration increased, the (100) ZnO plane diminished, and ZnOHF, Zn­(OH)_2_ and/or Zn­(OF)_2_ plane peaks dominated. Crystallinity was highly dependent on fluorine incorporation. In the XRD analysis, one of the extra peaks observed was tentatively assigned to ZnOF_2_ based on peak matching using the QualX software and the Crystallography Open Database (COD). However, ZnOF_2_ is a relatively unstable and rarely reported phase under standard ALD conditions. Its presence in the fluorine-doped ZnO (FZO) films may arise due to localized stoichiometric fluctuations at high fluorine concentrations or from metastable surface interactions during deposition.

**Figure 6:**
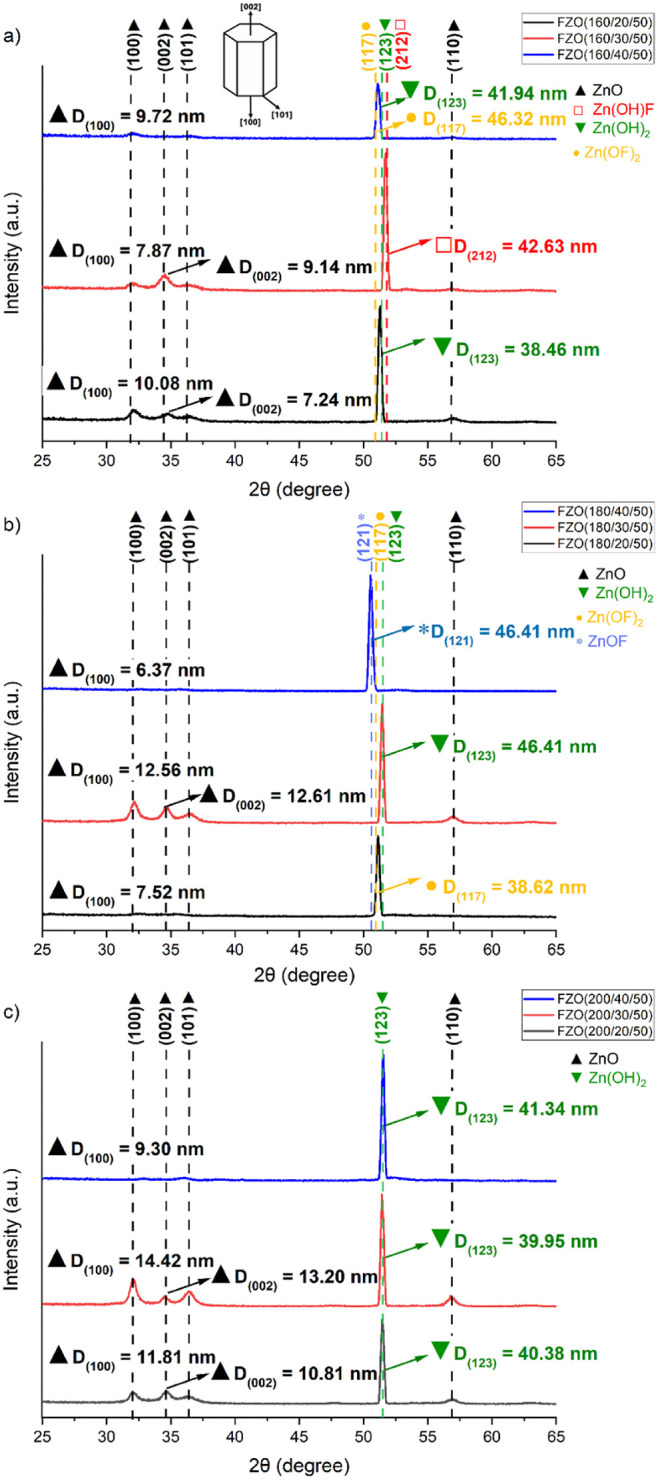
XRD patterns for samples with increasing NH_4_F/DI water concentrations (20%, 30%, and 40%, from bottom to top) grown at **a)** 160 °C, **b)** 180 °C, and **c)** 200 °C.

To support this assignment, F 1s and O 1s signals were detected in the XPS spectra, indicating the coexistence of fluorine and oxygen species. Nevertheless, because of the complexity of mixed phases and the overlapping nature of XRD peaks in Zn-based oxides and oxyfluorides, this assignment remains tentative.

At the 160 °C growth temperature, increasing NH_4_F/DI concentration systematically modified the crystalline structure of the FZO films. As the NH_4_F concentration rose from 20% to 30%, both ZnO (100) and (002) diffraction peaks diminished in intensity due to excessive fluorine incorporation. During this transition, the (100) plane grain size decreased from 10.08 to 7.87 nm, while the (002) plane grain size increased from 7.24 to 9.14 nm, indicating preferential *c*-axis growth enhancement. Concurrently, the dominant secondary phase shifted from Zn­(OH)_2_ (observed as the (123) plane peak at 20% concentration) to ZnOHF (appearing as the (212) plane peak at 30% concentration), demonstrating fluorine’s influence on crystal phase formation.

Further increasing the NH_4_F concentration to 40% reversed these trends, with the (100) plane grain size expanding to 9.72 nm, while the (002) plane grain size decreased. This concentration also produced mixed secondary phases, evidenced by the emergence of Zn­(OH)_2_ ((123) plane at 51.14°) diffraction peaks. The phase evolution from the ZnOHF to Zn­(OH)_2_ system correlates with suppressed *c*-axis growth in the (002) direction and promoted lateral expansion in the (100) direction, suggesting fluorine-mediated modification of the ZnO growth kinetics.

At the 180 °C growth temperature (Figure 6b), the crystalline evolution displayed distinct concentration-dependent behavior. As the NH_4_F concentration increased from 20% to 30%, both the (100) and (002) ZnO peaks exhibited grain size expansion, indicating enhanced crystallinity. This improvement correlates with the appearance of Zn­(OH)_2_, which stabilizes the ZnO lattice and promotes larger grain formation. However, further increasing the concentration to 40% reversed these trends. The (100) plane grain size decreased sharply from 12.56 to 6.37 nm, while the (002) peak diminished significantly. This degradation coincides with the transition from Zn­(OH)_2_ to ZnOF phases, where the latter introduces structural defects that inhibit proper ZnO crystallization. The ZnOF phase appears to disrupt the crystalline order, leading to deteriorated film quality at higher doping concentrations.

At the 200 °C growth temperature (Figure 6c), all samples (20–40% NH_4_F/DI) exhibited dominant (123) Zn­(OH)_2_ diffraction peaks. In the 20–30% concentration range, both ZnO crystallite dimensions increased significantly, with the (100) plane grain size expanding from 11.81 to 14.42 nm and the (002) plane growing from 10.81 to 13.20 nm. Concurrently, the Zn­(OH)_2_ (123) grain size decreased slightly from 40.38 to 39.95 nm, suggesting that moderate fluorine incorporation (≤30%) reduces structural defects while promoting balanced growth along both the lateral (100) and *c*-axis (002) directions. However, further increasing the NH_4_F concentration to 40% reversed these trends: the ZnO (100) grain size decreased sharply to 9.30 nm, while the (002) orientation diminished completely. This degradation coincided with Zn­(OH)_2_ grain growth to 41.34 nm, indicating that excessive fluorine doping (>30%) disrupts ZnO crystallization through the inhibition of *c*-axis growth and destabilization of ZnO lattice planes.

The chemical bonding and compositional analysis, as determined by HR XPS (F 1s peaks) and CASA XPS quantification (O 1s and Zn 2p3/2 regions), revealed significant concentration-dependent trends across the 160–200 °C growth temperature range (Figure 7). At 160 °C, the surface fluorine concentration exhibited unexpected behavior, with the 20% NH_4_F sample showing higher fluorine content (5.41 at. % F) than the 30% sample (3.64 at. % F), suggesting preferential fluorine segregation to the surface at lower precursor concentrations. The 40% NH_4_F sample demonstrated extreme surface fluorination, reaching 27.05 at. % F.

**Figure 7:**
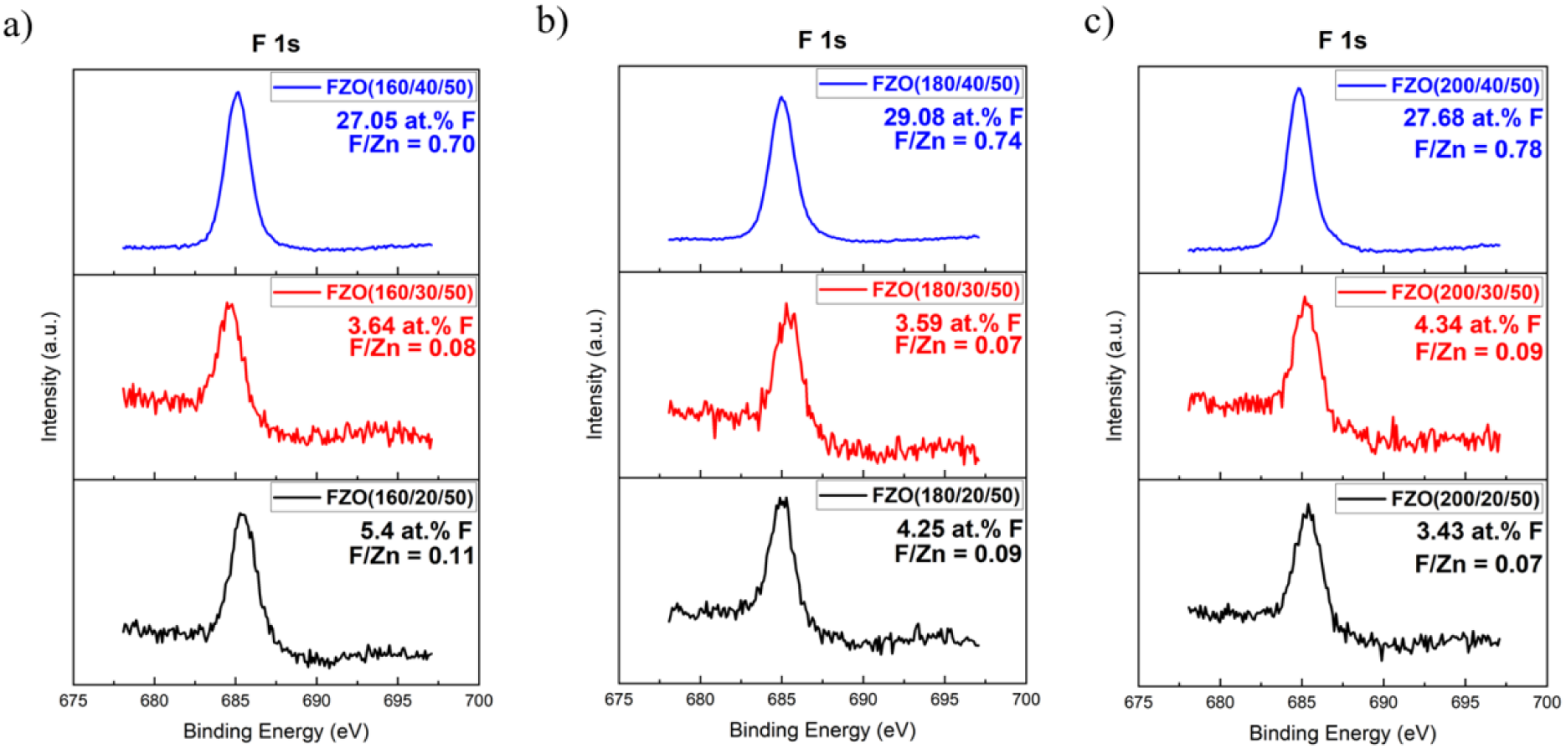
HR-XPS F 1s surface measurement peaks for samples with increasing NH_4_F/DI water concentrations (20%, 30%, and 40%, from bottom to top) grown at **a)** 160 °C, **c)** 180 °C, and **e)** 200 °C.

Chemical state analysis showed distinct bonding configurations at different concentrations. In the 20% NH_4_F samples, fluorine preferentially occupied oxygen vacancies and interstitial sites, leading to increased M-O and Zn^2+^ bonding while decreasing oxygen vacancies (V_o_) and zinc (Zn) content. In contrast, the 40% NH_4_F samples exhibited a dramatic transformation of the bonding environment, characterized by increased OH and ZnOH formation, with fluorine now bonding to both oxygen vacancies and zinc interstitials. This shift resulted in the predominance of Zn–OH complexes over the standard ZnO coordination.

Depth profiling analysis (Figure 8) provided crucial context, revealing that the bulk composition followed more conventional concentration trends compared with the surface measurements. The observed surface enrichment effects, particularly the inverse fluorine concentration relationship between the 20% and 30% samples, suggest a concentration-dependent saturation mechanism where moderate doping levels facilitate uniform bulk incorporation, while lower concentrations promote surface segregation.

**Figure 8:**
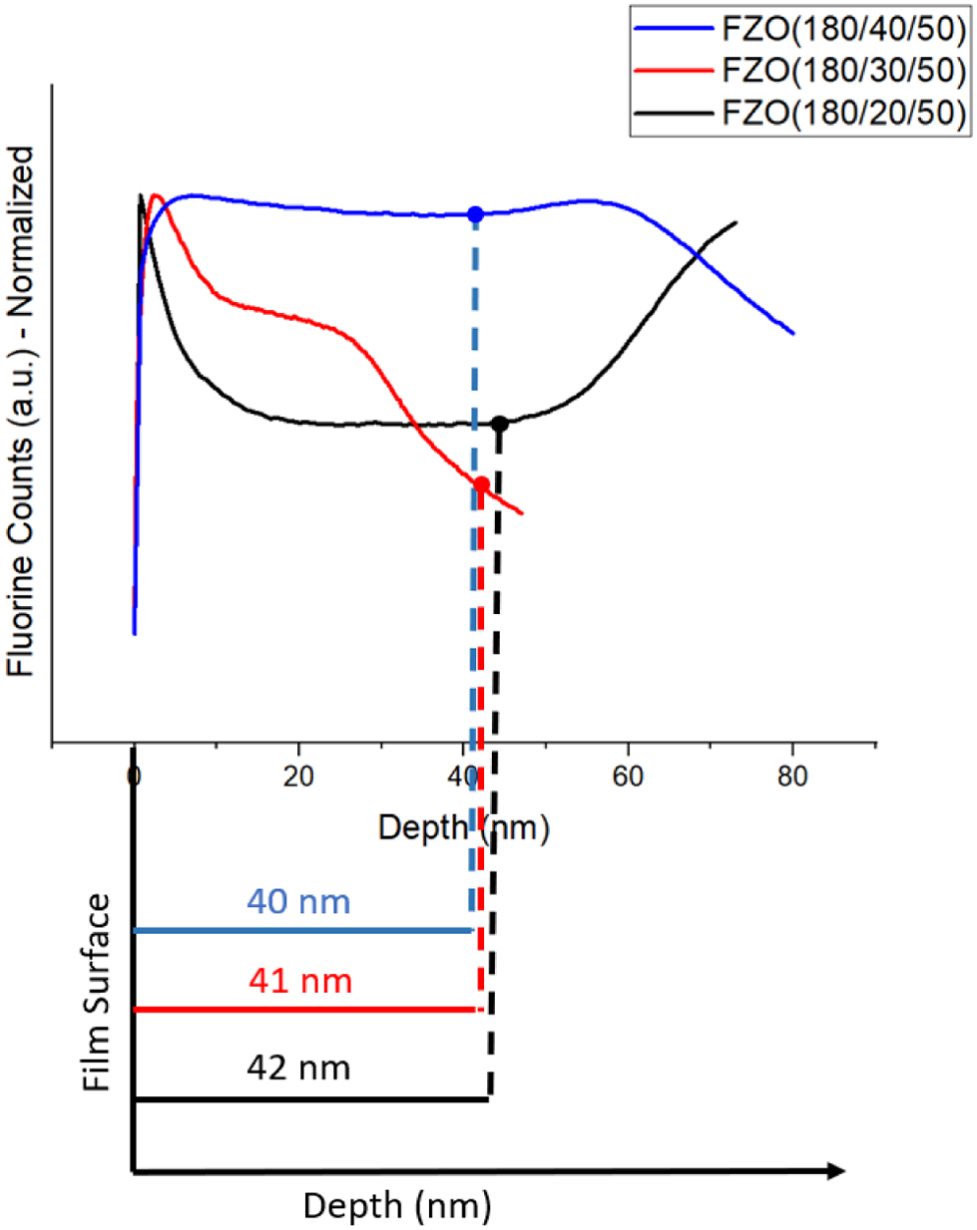
SIMS depth analysis for samples with increasing NH_4_F/DI water concentrations of 20%, 30%, and 40% at a growth temperature of 180 °C and a canister temperature of 50 °C. The fluorine counts along the film depth are normalized with respect to the film surface.

Consistent with the 160 °C growth results, films deposited at 180 °C with 20% NH_4_F/DI concentration exhibited higher surface fluorine content (4.25 at%) than their 30% counterparts (3.59 at%), while the 40% samples showed extreme surface fluorination (29.08 at%). Analysis of the O 1s and Zn 2p3/2 regions (Figure 7d) revealed systematic bonding changes across concentrations. When decreasing from 30% to 20% NH_4_F/DI, we observed reductions in M-O%, Zn%, and Zn^2+^ content, alongside increases in oxygen vacancy (V_o_%) and ZnOH% concentrations. In these 20% samples, fluorine exhibited a preference for bonding with zinc interstitials and zinc ions rather than occupying oxygen vacancy sites.

The transition from 20% to 40% concentration produced opposite trends, with M-O%, V_o_%, and ZnOH% decreasing, while OH%, Zn%, and Zn^2+^ content increased. At 40% concentration, fluorine anions predominantly occupied oxygen vacancies while also bonding with zinc interstitials and zinc ions.

For 200 °C-grown samples, the 40% NH_4_F/DI mixture again caused excessive surface fluorination, though, unlike the lower temperature samples, the 30% concentration showed higher fluorine content than the 20% samples. Increasing from 20% to 30% concentration led to decreased OH% and ZnOH%, with slight increases in V_o_%, Zn%, and Zn^2+^ content. In these 30% samples, fluorine preferentially bonded with zinc ions rather than filling oxygen vacancies. Further increasing to 40% concentration reduced M-O% and Zn^2+^ while increasing V_o_% and Zn%, with minimal changes to OH% and ZnOH% content. At this highest concentration, fluorine maintained its preference for bonding with zinc ions over oxygen vacancy occupation.

Figure 8 presents the SIMS depth profiles of fluorine concentration for 180 °C-grown FZO films with varying NH_4_F/DI concentrations (20%, 30%, and 40%). The fluorine signals were normalized to their respective surface concentrations, while film thicknesses were determined from the onset of the silicon substrate signal (marked by dots).

While the 20% concentration sample showed higher initial surface fluorination, the 30% and 40% samples exhibited a significantly more uniform fluorine distribution throughout the film depth. This improved homogeneity in higher-concentration samples suggests more effective bulk incorporation of fluorine atoms during ALD growth, contrasting with the surface segregation tendency observed at lower concentrations.

The optical transmittance characteristics of FZO films grown at different temperatures (160 °C, 180 °C, and 200 °C) with varying NH_4_F/DI concentrations (20%, 30%, and 40%) are presented in Figure 9. For films grown at 160 °C, the average visible-range transmittance (400–700 nm) measured 87.7%, 89.8%, and 88.2% for 20%, 30%, and 40% concentrations, respectively. The 180 °C-grown samples showed similar trends, with visible-range average transmittance values of 85.7%, 89.4%, and 85.7% for the corresponding concentrations. Notably, the 200 °C-grown films exhibited distinct behavior, with visible-range average transmittance values of 85.6%, 89.2%, and an exceptional 96.5% for 20%, 30%, and 40% concentrations, respectively.

**Figure 9:**
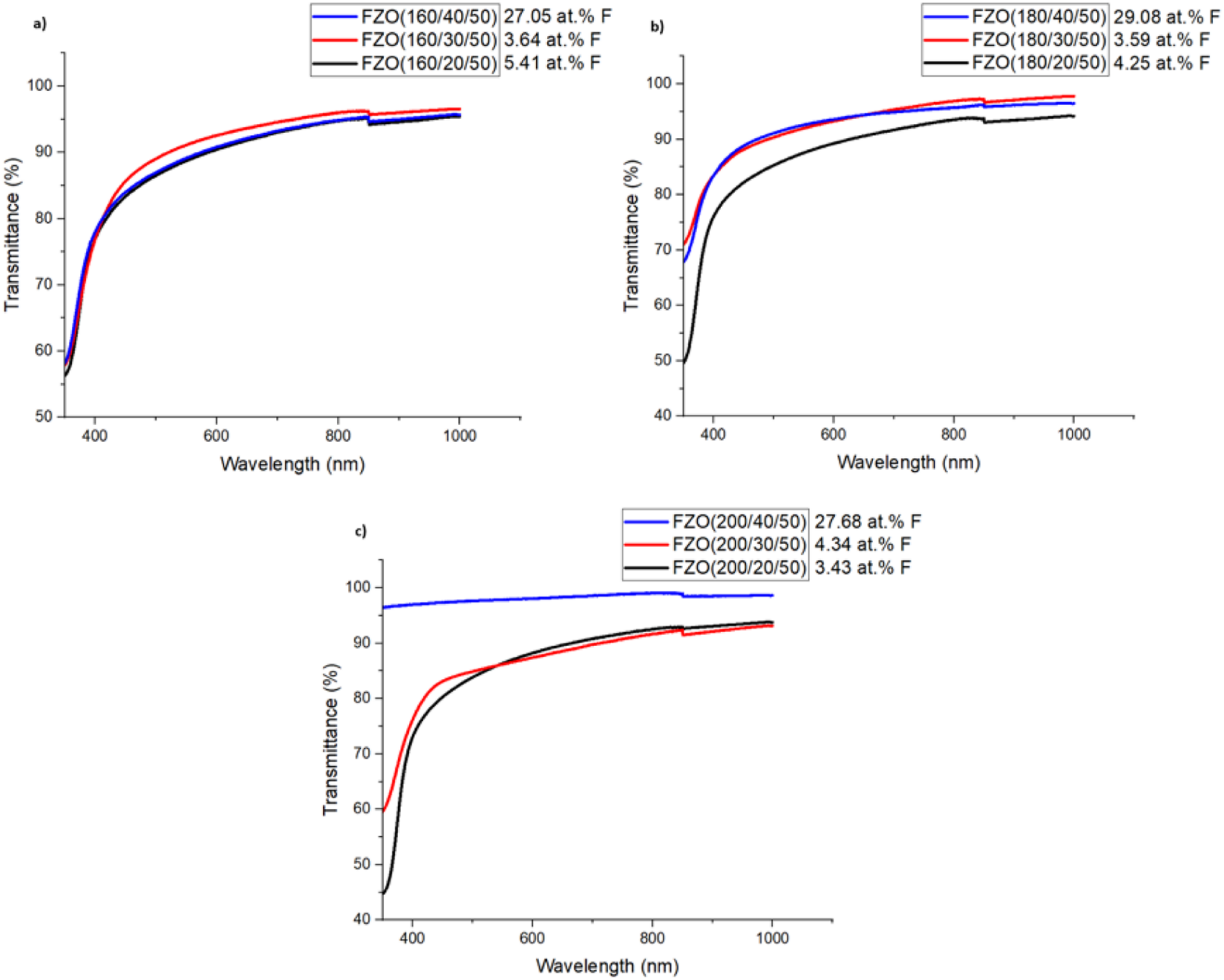
Transmittance percentages for samples with varying NH_4_F/DI water concentrations (20%, 30%, and 40%) grown at **a)** 160 °C, **b)** 180 °C, and **c)** 200 °C.

The unusually high transmittance (96.5%) observed for the 200 °C–40% sample represents an outlier case, attributable to extreme surface fluorination (27.68 at. % F) and significantly reduced film thickness resulting from synergistic thermal and chemical etching effects. Under standard conditions, the visible-range average transmittance values remained within the 85.6–89.8% range, with reductions primarily caused by two mechanisms: increased light scattering from excess zinc content through defect formation,^47–49^ and enhanced photon scattering from fluorine-induced crystal defects at moderate temperatures.^54^

A consistent pattern emerged where 30% NH_4_F/DI concentration yielded optimal visible-range average transmittance across all growth temperatures (89.8% at 160 °C, 89.4% at 180 °C, and 89.2% at 200 °C). This suggests that the 30% concentration represents the ideal balance between stoichiometric control and defect minimization for optical applications.

#### 4.3.1 Mechanism Interpretation

The incorporation of fluorine into ZnO films was found to be critically dependent on the growth temperature. Notably, surface analysis revealed that 20% NH_4_F/DI mixtures yielded higher fluorine concentrations than 30% mixtures at 160 and 180 °C, a phenomenon attributed to preferential fluorine–carbon surface bonding. Temperature also governed the underlying incorporation mechanisms; at lower temperatures (160 °C), fluorine favored occupation of oxygen vacancies and bonding to zinc interstitials, while at a higher temperature (200 °C), direct bonding with zinc atoms was the predominant mechanism.

Although a more homogeneous concentration gradient was obtained for higher concentration precursor samples, the analysis revealed that NH_4_F concentrations above 30% had a deleterious impact on the material. Specifically, higher concentrations promoted excessive fluorine incorporation, which manifested as increased point defects (e.g., fluorine interstitials), the creation of scattering centers at grain boundaries, and the precipitation of secondary phases (Zn­(OH)_2_, ZnOHF), ultimately degrading crystallinity. Therefore, an NH_4_F concentration of ≤20% is recommended to optimize material quality by limiting fluorine uptake and mitigating defect formation.

### 4.4 Primary Electrical Properties Results of FZO Films

The electrical performance of FZO films as TCOs is critically dependent on both the free carrier concentration and crystalline quality. Crystal defects function as trap states and scattering centers, simultaneously reducing the charge carrier density through compensation mechanisms and decreasing carrier mobility through enhanced scattering. Fluorine concentration plays a dual role in these electrical properties by significantly influencing crystallinity.

At optimal doping concentrations below 1.5 atomic percent fluorine (<1.5 at. % F), fluorine ions effectively passivate oxygen-related defects while enhancing ZnO crystallinity, as demonstrated in previous studies.^47,48^ This improvement in crystallinity is accompanied by increased grain size,^54^ which reduces grain boundary area and consequently improves both carrier concentration and mobility, leading to enhanced overall conductivity.^19^ However, when fluorine concentration exceeds this optimal threshold (>1.5 at. %), several detrimental effects emerge due to the ionic radius difference between fluorine (0.136 nm) and oxygen (0.140 nm). The shorter Zn–F bond length (1.93 Å) compared to Zn–O (1.97 Å) introduces lattice strain that disrupts crystal growth, ultimately decreasing grain size, degrading crystallinity, and reducing conductivity.^47,49,55^

Excessive fluorination negatively impacts electrical properties through multiple mechanisms, including the creation of additional scattering centers, the formation of undesirable secondary phases, the erosion of preferred ZnO crystal planes, and the compensation of n-type doping through Zn-NH_3_^+^ interactions. These combined effects lead to increased film resistivity at higher fluorine doping levels.^53,55,56^

Therefore, among the 11 process conditions explored for fluorine incorporation investigation (see Table S1), the FZO­(180/30/40) sample with 2.62 at. % F exhibited the best electrical performance, achieving a resistivity of 2.14 Ω·cm, carrier concentration of 1.24 × 10^18^ cm^–3^, and mobility of 2.36 cm^2^/(V·s), along with an excellent average visible transmittance of 89.4%. These results compare well with literature values for CVD-grown FZO films with comparable fluorine concentrations (2–3 at. % F), which typically show higher resistivity (18.57 Ω·cm) and lower transmittance (84%).^57^ While ALD-grown films using HF precursors have demonstrated higher resistivity (3.98 × 10^–3^ Ω·cm) at lower doping concentrations (0.2 at. % F), our NH_4_F-based process achieves better overall performance at higher doping levels, highlighting the advantages of precursor selection and growth optimization in balancing electrical and optical properties. A detailed, dedicated electrical optimization of fluorine doping with NH_4_F is under investigation.

## 5 Conclusion

This study successfully demonstrated the NH_4_F/DI mixture as a fluorine doping source for ALD growth of FZO films. Through systematic optimization of deposition parameters—including growth temperature (160–200 °C), NH_4_F/DI concentration (20–40%), and precursor canister temperature (RT, 40 °C, 50 °C)—we established several key findings regarding fluorine incorporation and its effects on film properties.

The fluorine doping concentration exhibited a direct correlation with the canister temperature, with higher temperatures promoting increased incorporation. However, excessive NH_4_F/water concentrations (above 30%) led to detrimental effects, including elevated fluorine content, generation of scattering centers (grain boundaries, fluorine interstitials), formation of undesirable secondary phases (Zn­(OH)_2_, ZnOHF), degradation of ZnO crystal planes, and inhibition of effective n-type doping through Zn-NH_3_^+^ interactions. These combined factors significantly degraded the electrical performance of overfluorinated films.

Notably, surface analysis revealed higher fluorine concentrations for 20% NH_4_F/DI mixtures compared to 30% at 160 and 180 °C growth temperatures, attributed to preferential fluorine–carbon bonding at the surface. Furthermore, deposition temperature strongly influenced incorporation mechanisms—at 160 °C, fluorine preferentially occupied oxygen vacancies and bonded with zinc interstitials, while at 200 °C, bonding with zinc atoms dominated.

Among the 11 process conditions explored, optimal electrical properties were achieved for a growth temperature of 180 °C with 30% NH_4_F/DI and a canister temperature of 40 °C, yielding films with a 2.62 at. % fluorine concentration that exhibited resistivity of 2.14 Ω·cm, carrier concentration of 1.24 × 10^18^ cm^–3^, and Hall mobility of 2.36 cm^2^/V·s. All FZO films maintained excellent optical transparency with an average visible-range (400–700 nm) transmittanceof ≥80%.

Comparative analysis with literature values (typically 10^–4^ Ω·cm resistivity at ∼1.5 at. % F) suggests that further optimization using lower NH_4_F/DI concentrations (10–20%) with elevated canister temperature (50 °C) could achieve superior performance. The observed temperature-dependent incorporation trends indicate that ideal NH_4_F/DI concentrations should be adjusted according to growth temperature—lower concentrations (≤10%) for higher temperatures (200 °C) and slightly higher concentrations for lower-temperature depositions.

## Supplementary Material



## Notes

Figure S1, a) The CASA XPS program calculations of O 1s and Zn 2p_3/2_ regions for corresponding at. % F for samples with increasing canister temperatures from bottom to top (RT, 40 °C, and 50 °C) at a growth temperature of 180 °C and 30% NH_4_F/H_2_O concentration, and the CASA XPS program calculations of O 1s and Zn 2p_3/2_ regions at **b)** 160 °C, **c)** 180 °C, and **d)** 200 °C for samples with increasing NH_4_F/DI water concentrations (20%, 30%, and 40% from bottom to top). Table S1. The Haacke figure of merit (Φ_TC_) was calculated using Φ_TC_ = T^10^/R_S_, where *T* is the average transmittance, in the 400–700 nm range (PDF)

## #

Air Liquide Laboratories, Electronics Customer Process, Yokosuka, Kanagawa, Japan
