# Supplementary material for: In Situ Fluorine Doping of FZO Films by Atomic Layer Deposition
Source: ACS Omega. 2026 Jul 19;11(30):44634–45. doi: 10.1021/acsomega.5c10192 (PMC13448994; doi:10.1021/acsomega.5c10192)
Supplement: Supplementary file 1 [file ao5c10192_si_001.pdf]

Supporting Information

In-situ Fluorine Doping of FZO films by Atomic Layer Deposition

Meryem Tunckanat[1,†] , Bilge Imer[2,1,*]

[1] Micro and Nanotechnology Department, Middle East Technical University, Ankara, 06800, Türkiye

† Current address: Air Liquide Laboratories, Electronics Customer Process, Yokosuka, Kanagawa, Japan

[2] Department of Metallurgical and Materials Engineering, Middle East Technical University, Ankara, 06800, Türkiye

* Corresponding author: bimer@metu.edu.tr

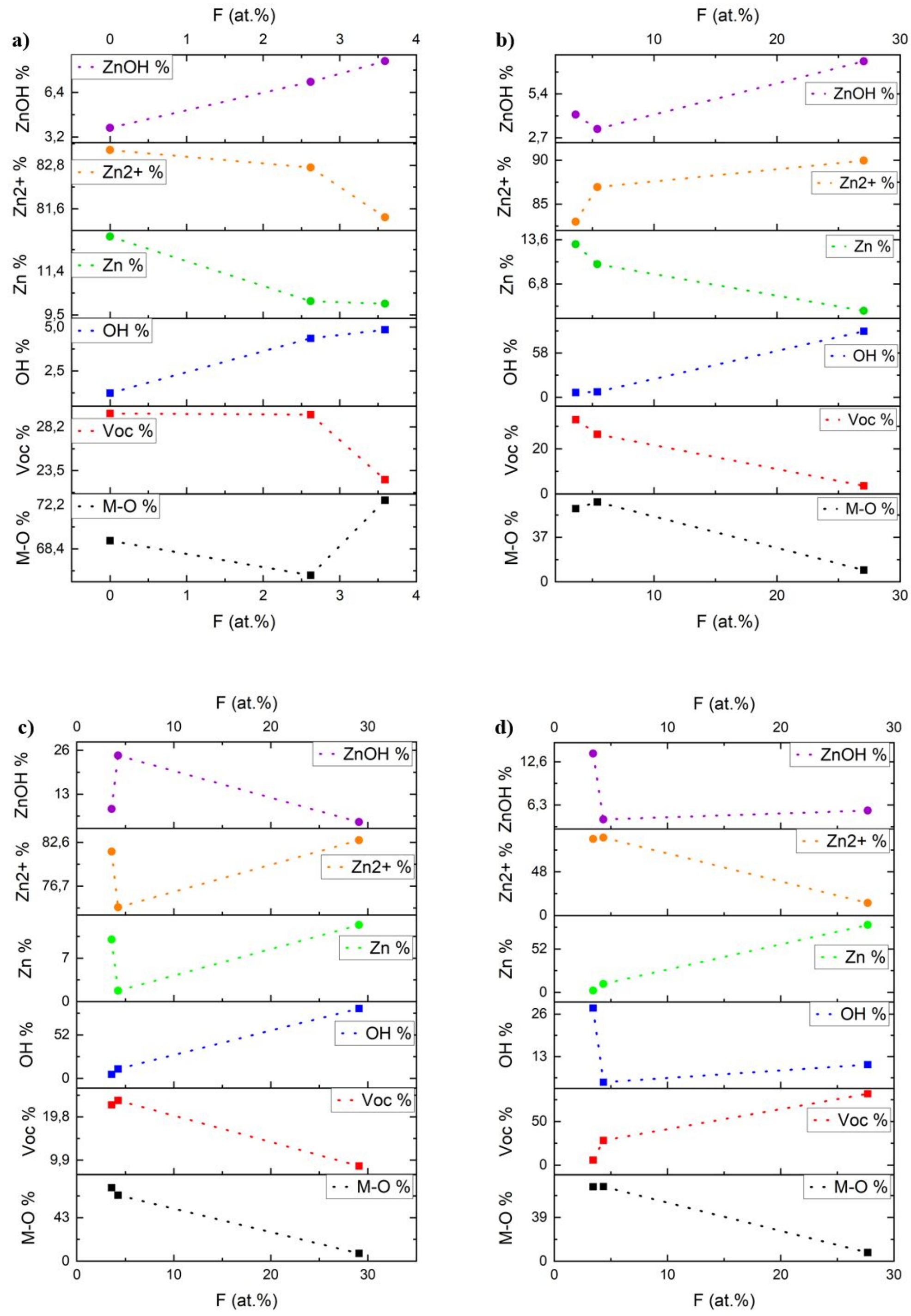

Figure S1. a) the CASA XPS program calculations of O1s and $Zn2p_{3/2}$ regions for corresponding at. % F for samples with increasing canister temperatures from bottom to top (RT, 40°C and 50°C) at growth temperature of 180°C and 30% NH_4F/H_2O concentration, and the CASA XPS program calculations of O1s and $Zn2p_{3/2}$ regions at **b)** 160°C, **c)** 180°C, and **d)** 200°C for samples with increasing NH_4F/DI water concentrations (20%, 30% and 40% from bottom to top)

Table S1. The Haacke figure of merit (Φ_{TC}) was calculated using $\Phi_{TC} = T^{10}/R_S$, where T is the average transmittance, in the 400–700 nm range.

Sample ID	Thickness measured from Si substrate peak by SIMS (nm)	Sheet Resistance (Ω·/sq.)	Φ_{TC}	Avr. Transmittance (%) 400-700 nm
**FZO(160/20/50)**	39	8.08E+06	3.33E-08	87.7
**FZO(160/30/50)**	38	2.12E+09	1.61E-10	89.8
**FZO(160/40/50)**	40	3.23E+09	8.82E-11	88.2
**FZO(180/20/50)**	42	2.61E+07	8.18E-09	85.7
**FZO(180/30/RT)**	65	2.43E+09	5.00E-11	81.13
**FZO(180/30/40)**	38	2.23E+05	1.5E-6	89.4
**FZO(180/30/50)**	41	5.51E+08	5.92E-10	85.7
**FZO(180/40/50)**	40	1.85E+09	1.15E-10	85.6
**FZO(200/20/50)**	42	9.47E+07	2.23E-09	89.2
**FZO(200/30/50)**	40	1.26E+09	2.54E-10	89.1
**FZO(200/40/50)**	35	8.55E+08	8.19E-10	96.5
